# Molecular characterisation of human hepatitis E virus from Italy: comparative analysis of five reverse transcription-PCR assays

**DOI:** 10.1186/1743-422X-11-72

**Published:** 2014-04-22

**Authors:** Giuseppina La Rosa, Marta Fratini, Michele Muscillo, Marcello Iaconelli, Stefania Taffon, Michele Equestre, Paola Chionne, Elisabetta Madonna, Giulio Pisani, Roberto Bruni, Anna Rita Ciccaglione

**Affiliations:** 1 Department of Environment and Primary Prevention, Istituto Superiore di Sanità, Viale Regina Elena 299, 00161 Rome, Italy; 2 Department of Infectious, Parasitic and Immune-Mediated Diseases, Istituto Superiore di Sanità, Viale Regina Elena 299, 00161 Rome, Italy; 3 Department of Cell Biology and Neurosciences, Istituto Superiore di Sanità, Rome, Italy; 4 Center for Immunobiologicals Research and Evaluation, Istituto Superiore di Sanità, Rome, Italy

**Keywords:** Hepatitis E virus, RT-PCR assays, Molecular characterization, Sequencing, Genotyping

## Abstract

**Background:**

Hepatitis E (HEV) is an important public-health concern as a major cause of enterically transmitted hepatitis worldwide. In industrialised countries it is considered rare, and largely confined to travellers returning from endemic areas. However, autochthonous (locally acquired) HEV infection is also emerging in these regions. The infection is caused by different genotypes, depending on whether it is travel-related or autochthonous. Conventional RT-PCR followed by sequencing of PCR products can identify HEV genotype and, depending on the region, the subtype, thus helping in defining the origin of infection and tracing the source of contamination.

**Methods:**

We re-analysed a collection of serum samples previously confirmed as hepatitis E positive by anti-HEV IgM and IgG assays as well as by Real-Time PCR, with the aim to compare the performances of five different broad range RT-PCR assays that could be provided for molecular characterisation of HEV. This approach is certainly valuable to investigate the molecular epidemiology of acute hepatitis E in countries where co-circulation of different genotypes occurs, like Italy.

**Results:**

Samples were analyzed by five assays targeting the ORF1, ORF2, and ORF2/3 regions. The sensitivity of these assays varied significantly, depending on the target region. Only 46% of samples tested positive by nested PCR; moreover, no single method was able to detect all positive samples. Most sequences originated from patients who had travelled to endemic areas (genotype 1), while the minority originated from Italian patients with no travel history (genotype 3).

**Conclusion:**

Broad range methods for molecular characterization of HEV still need to be improved to detect all circulating strains.

## Background

Hepatitis E virus (HEV) is a single-strand positive-sense RNA virus, classified as the sole member of the genus *Hepevirus* in the family *Hepeviridae*. HEV is divided into four genotypes, all belonging to a single serotype, further divided into a total of 24 subtypes [1,2]. Genotypes 1 (G1) and 2 (G2) are associated with human illness, while genotype 3 (G3) and 4 (G4) are animal strains which are occasionally transferred to humans. The HEV genome has three open reading frames (ORFs): ORF1 encodes the non-structural polyprotein that contains various functional units: methyl transferase, papain-like cysteine protease, RNA helicase, and RNA dependent RNA polymerase. ORF2 encodes the viral capsid protein and ORF3 a small regulatory phosphoprotein [3].

The infection may vary in severity from inapparent to fulminant liver failure and death. The mortality rate is higher than hepatitis A, ranging between 1% and 4% [4]; the death rate approaches 25% in pregnant woman.

In endemic regions (Asia, Africa, Middle East) hepatitis E occurs in epidemic forms, while in industrialized countries HEV occurs sporadically and both travel related and autochthonous infections are reported.

Data from the national surveillance system for acute hepatitis indicated that in Italy HEV is responsible for about 10% of the acute cases [5]; this percentage is most probably underestimated since systematic testing of non-A-C cases for hepatitis E is not routinely performed. A long term prospective study from Italy, by Romanò and co-workers, conducted over 15 years, revealed that 20.6% of non-A-C patients had acute HEV infection [6]. Of these, most cases were imported and caused by genotype 1 while some autochthonous cases were caused by genotype 3. Similar results were described in another Italian study in which most infections, due to genotype 1, were associated with travel to endemic areas (Bangladesh, India and Pakistan), while the remaining infections, due to genotype 3, were autochthonous, presumably linked to exposure to raw seafood, pork liver sausages and wild boar [7]. A recent paper by Garbuglia and coworkers showed that HEV genotype 4, endemic among humans in China, Japan, India, and Indonesia, is also circulating in Italy in non travellers to disease-endemic areas [8].

The objective of the present study was to evaluate different nested RT-PCR assays potentially useful for molecular characterisation of circulating HEV strains. This approach is certainly valuable to investigate the molecular epidemiology of acute hepatitis E in countries where co-circulation of different genotypes occurs, like Italy.

The great genetic diversity of HEV viruses (four major genotypes - G1 to G4 - and several subtypes within genotypes) makes it very difficult to design universal primers for sensitive detection of all genotypes of HEV by RT-PCR. It was reported that the inter-genotype diversity over the entire genome of HEV is 23.6–27.7%, and the intra-genotype diversities of genotypes 1, 3, and 4 are as high as 11.8, 19.3, and 17.0%, respectively [8]. A variety of assays have been published in scientific literature, targeting the three highly conserved regions among HEV genomes: the 5′-terminal part of ORF1, the ORF2/ORF3 overlapping region, and the central portion of ORF2. In this study we compared five widely used RT-PCR assays (nested type) available in literature, targeting the three conserved regions, in order to evaluate their sensitivity.

## Materials and methods

We re-analysed a collection of serum samples previously confirmed as HEV-positive by anti-HEV IgM and IgG assays as well as by Real-Time PCR [9]. Samples were collected in 17 infectious disease units, situated in 11 of the 20 regions of Italy, from 2004 to 2013. Written informed consent for participation in the study was obtained from participants or, where participants are children, a parent or guardian. The study was approved by the Ethics Committee of the Italian National Institute of Health. A total of 24 samples positive for three markers (IgM, IgG and HEV RNA) were chosen for this investigation. HEV RNA was extracted from 200 μL serum samples using silica columns provided with the QIAamp MinElute Virus Spin kit (Qiagen, Hilden, Germany) according to the manufacturer’s instructions, aliquoted, stored at -80°C and thawed only once.

The extracted RNAs were analyzed by RT-PCR with nested strategy, using five broad range HEV-specific sets of primers targeting the ORF1, the ORF2, and the ORF2/3 regions, here called Method A, B, C, D, E.

**Figure 1:**
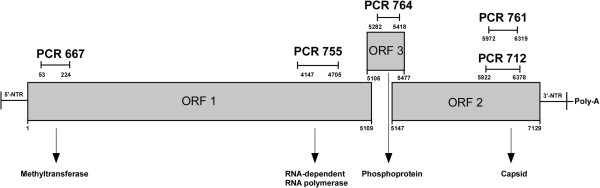
**Genome organization of HEV along with target region for the five assays**[2]**.** Positions are based on GenBank sequence accession no. M73218.

Primers used in this study as well as target regions along HEV genome are shown in Table 1 and Figure 1, respectively.

**Table 1:** Primer and PCRs used in this study

**Method**	**Target region**	**Primer ID**	**Sequence (5′–3′)**	**Product length (bp)**	Primer position (5′–3′)*
Method A	ORF1	1679	CCAYCAGTTYATHAAGGCTCC	348	36-56
		1680	TACCAVCGCTGRACRTC		383-367
		1681	CTCCTGGCRTYACWACTGC	172	53-71
		1682	GGRTGRTTCCAIARVACYTC		224-205
Method B	ORF1	1829	ACATTTGAATTATCTGACATTGTGCA	1076	3880-3905
		1828	ACACACATCTGAGCTACATTCGTGAG		4955-4930
		1830	GACGTGTCCAGGATCACCTTCTTC	559	4147-4170
		1831	ACTCACTGCAAAGCACTATCGAAT		4705-4682
Method C	ORF2	1722	CAAGGHTGGCGYTCKGTTGAGAC	506	5912-5934
		1723	CCCTTRTCCTGCTGAGCRTTCTC		6417-6395
		1724	GYTCKGTTGAGACCWCBGGBGT	457	5922-5943
		1725	TTMACWGTCRGCTCGCCATTGGC		6378-6356
Method D	ORF2	1837	AATTATGCYCAGTAYCGRGTTG	731	5687-5708
		1838	CCCTTRTCYTGCTGMGCATTCTC		6417-6395
		1839	GTWATGCTYTGCATWCATGGCT	348	5972-5993
		1840	AGCCGACGAAATCAATTCTGTC		6319-6298
Method E	ORF2/ORF3	1847	GCRGTGGTTTCTGGGGTGAC	164	5259-5278
		1848	CTGGGMYTGGTCDCGCCAAG		5422-5403
		1849	GYTGATTCTCAGCCCTTCGC	137	5282-5301
		1850	GMYTGGTCDCGCCAAGHGGA		5418-5399

*Primer positions are based on GenBank sequence accession no. M73218.

**Method A:** an assay targeting the Methyltransferase (MTase) gene in the ORF1. Primers were developed by Fogeda and collaborators and amplify a 172 bp region within the ORF1 [10].

**Method B**: an assay targeting the RNA-dependent RNA polymerase (RdRp) gene in the ORF1. Primers were originally engineered by Aggarwal [11] and amplify a 559 bp long segment in the RdRp gene.

**Method C**: an assay targeting the capsid gene in the ORF2. This method was developed by Shrestha and coworkers [12], and later slightly modified by our group [7]. The expected product of the nested PCR is 457 bp. This assay is able to determine HEV genotypes as well as subtypes.

**Method D:** an assay targeting the capsid gene in the ORF2. Primers were developed by Huang and collaborators in order to amplify a 348 bp fragment within the ORF2 region [13].

**Method E:** an assay targeting the ORF2/ORF3. This method amplifies a 137 bp region and was proved to be useful for detection of hepatitis E virus strains with significant sequence divergence [8].

PCR amplification was performed in a 25 μl reaction volume using the MyTaq™ One-Step RT-PCR Kit (Bioline). Amplification conditions were as described by the authors. PCR products were separated on a 1% agarose gel stained with GelRed. Following amplification, PCR products of the expected size were purified using a Montage PCRm96 Micro-well Filter Plate (Millipore). Bidirectional sequencing was performed using the above-mentioned amplification primers and the GenomeLab™ DTCS Quick Start Kit (Beckman Coulter, Inc., Fullerton, CA) according to the manufacturers protocol. Sequencing reactions were run on an automated DNA sequencer (Beckman Coulter, Inc., Fullerton, CA). Phylogenetic analysis was then performed to assess the genetic relationships among the different sequences and between the sequences of the samples and those of the prototype strains. Phylogenetic analysis was performed using MEGA software version 5.2.1. Nucleotide sequences were aligned using the Clustal W algorithm [14]. The phylogenetic tree was constructed using the Maximum Likelihood method based on the Kimura 2-parameter model, integrated into the MEGA software [15]. The robustness of the clustering results was assessed by bootstrap resampling (1000 replicates). The same topology was obtained when reconstruction was performed using BEAST (Bayesian Evolutionary Analysis by Sampling Trees) program [16] for Bayesian MCMC (Markov Chain Monte Carlo) analysis of molecular sequences (data not shown).

Consensus sequences, generated by aligning the forward and reverse sequences for each PCR products, were submitted to the EMBL Nucleotide Sequence Database using the Webin Submission Tool available at http://www.ebi.ac.uk/ena/about/submit_and_update.

## Results

Eleven (46%) of the 24 serum samples were positive for HEV RNA by one or more of the PCR assays used. Table 2 shows, besides results of nested PCR assays, the quantitative Real time PCR data obtained previously [9], where "+" means 250 to 2,500 copies/mL, "++" 2,500 < copies/mL <25,000, and "+ + +": >25,000 copies/mL.

**Table 2:** PCR results using five different nested assays

**Patient code**	**Real-Time PCR**^***a***^	**RT-PCR/nestedPCR**					**Country of Origin**^**b**^
		**Method A (ORF1, MTase)**	**Method B (ORF1, RdRp)**	**Method C (ORF2, capsid)**	**Method D (ORF2, capsid)**	**Method E (ORF2/ORF3)**	
8	+ + +	Genotype 1	Genotype 1	Genotype 1a	Genotype 1a	Genotype 1	Italy (India)
10	+	-		-	-	-	Ethiopia
12	+ + +	-		-	-	Genotype 1	India
16	+ +	Genotype 1		Genotype 1a	Genotype 1a	Genotype 1	Bangladesh
19	+ +	-		-	-	-	Bangladesh
22	+ +	-	Genotype 1	Genotype 1a	Genotype 1a	Genotype 1	Italy (India)
29	+	-		-	-	-	Bangladesh
33	+	-		-	-	-	Italy
34	+	-		-	-	-	Italy
43	+ +	Genotype 1		-	-	-	Bangladesh
47	+ +	-		-	-	-	Bangladesh
48	+ +	-	Genotype 1	-	-	-	Bangladesh
51	+ +	-		-	-	-	Italy
52	+	Genotype 1		Genotype 1a	-	-	Bangladesh
55	+ +	Genotype 1		-	-	-	India
56	+ +	-		-	-	-	Bangladesh
58	+ +	Genotype 1		-	-	-	Italy (Thailand)
61	+ +	-		-	-	-	Italy (India)
63	+ + +	-		-	-	-	Albania
69	+	Genotype 3		-	-	Genotype 3	Italy
71	+	-		-	-	-	Bangladesh
81	+	-		-	-	-	Italy
86	+	-		-	-	-	Italy
92	+	Genotype 3		-	-	-	Italy

^***a***^+ : 250 < copies/mL < 2,500; + + : 2,500 < copies/mL <25,000; + + +: >25,000 copies/mL.

^b^Travel to/from country of origin for foreign patients who travelled abroad in the last month. Travel to countries from endemic areas are indicated in brackets for Italian patients.

Method A (ORF1, MTase) was the more sensitive, with 8 out of 11 positive samples detected – six G1 and two G3. Method E (ORF2/3) detected 5 positives, of which four G1 and one G3. All the other methods detected only G1 (4 for Method C, and 3 for Methods B and D). We therefore detected a total of 9 G1 and 2 G3 positive samples by combining the five methods (Table 2 ). Only 4 of the 11 positive samples were detected in the ORF2, all belonging to G1, subtype G1a. The comparison of the sensitivities of the five assays is shown in Table 3.

**Table 3:** Sensitivities of PCR assays (detected samples by each method/total positive samples)

	**Method A**	**Method B**	**Method C**	**Method D**	**Method E**
Positive samples	8/11, 72%	3/11, 27%	4/11, 36%	3/11, 27%	5/11, 45%

G1 HEV was detected in serum samples from 4 Bangladeshi and 2 Indian patients, all returning from a trip to their country of origin shortly before the onset of symptoms, and in 3 Italian patients returning from recent trips to India or Thailand. The two patients with G3 were Italian, none of whom had travelled abroad recently nor had contact with recent travelers. Unfortunately, no information regarding other risk factors identified in this group is available.

Analysis of the data in regard to the viral load measured by PCR at sampling, showed, in general, a positive trend between the yield of RT-PCR positivity and the viral load, as shown in Table 4. However, some samples were not detected by the nested assay despite the high viral load ("+++"); on the other hand, some samples with low RNA quantity ("+") were detected. As for the relationship between the genotype and the viral load, we found that patients with G1 (travellers) displayed higher viral loads (7 samples "++" and 2 samples "+++") than patients (non-travellers) with G3 (2 samples "+"). Moreover, the overall yield of RT-PCR testing found among samples from travellers was twice higher than the yield obtained among the non-travellers (9/17, 53% vs. 2/7, 25%).

**Table 4:** PCR results compared to viral load measured at sampling

**Load (Real-time RT-PCR)**	**N**	**% positive (RT-PCR nested)**					
		**Method A**	**Method B**	**Method C**	**Method D**	**Method E**	**All**
+++	3	33	33	33	33	66	66
++	12	42	17	25	17	17	58
+	9	22	0	0	0	11	22
Total	24	33	13	17	13	21	46

**Figure 2:**
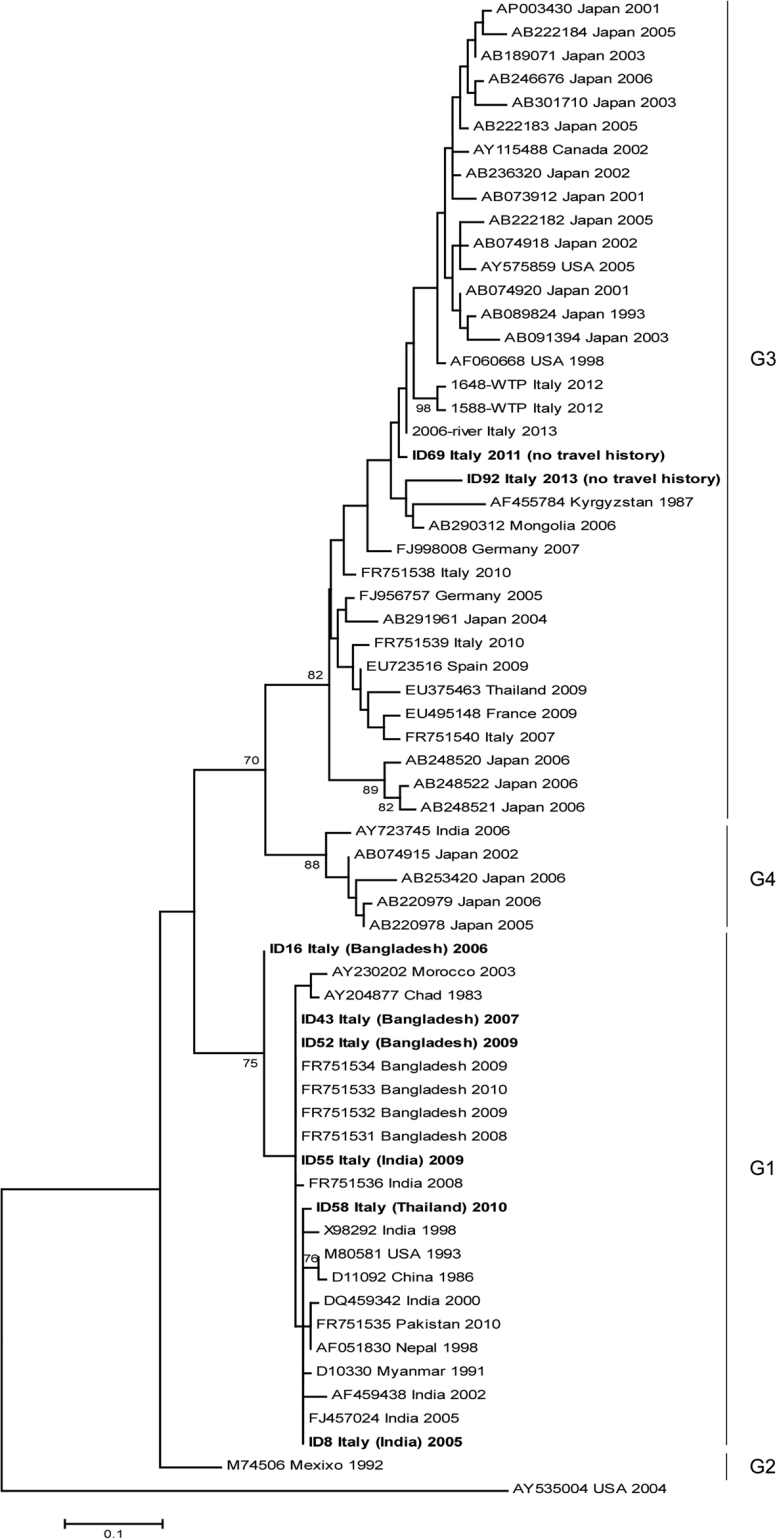
**Phylogenetic analysis in ORF1 (broad-range MTase assay).** HEV GenBank sequences are cited by their respective accession number followed by country and year of isolation. Study sequences are given in bold and are cited as follows: ID sample, geographical origin of the strain, and year; patient’s travel history is in brackets. Only bootstrap values greater than 70% are shown.

The results of the phylogenetic study performed on the sequences obtained using the broad-range MTase assay (ORF1) are presented in Figure 2. The tree was constructed by aligning the 8 sequences obtained from our amplification products, 53 HEV G1–G4 sequences from GenBank, and 3 environmental HEV sequences, detected in two sewage samples and one river sample in Italy by our group (unpublished data). An avian HEV isolate (AY535004) was included as an outgroup. The 65 sequences clustered into four main groups, corresponding to genotypes 1–4. The G1 cluster comprises strains from Asia and Africa as well as the following sequences obtained in the present study: ID8, 16, 43, 52, 55 and 58. The cluster also includes G1 strains identified in Italy in previous studies (FR751531 to FR751536) from Bangladeshi and Indian patients with acute HEV returning from a trip in their country of origin [6,7]. The G3 cluster includes strains from humans and animals (pigs, wild boar, wild deer and mongoose) detected worldwide, as well as the sequences ID69 and ID92 obtained in the present study, both identified from Italian patients with no travel history, of probable zoonotic origin. These sequences are similar to strains identified recently in sewage (ID1648 and 1588) and river samples (2006) in Italy by our group (unpublished data), and cluster with HEV sequences previously identified in serum from Italian patients with no travel history (Acc. No. FR751538- FR751540) [7]. The accession numbers for the sequences reported in this paper are: HG325846 to HG325862.

## Discussion

RNA is an important marker of acute HEV infection, especially during early stages, before the antibody response becomes evident. Serological testing alone may fail to diagnose acute infection, especially in immunocompromised patients, which justifies the use of molecular assays for diagnosis. Several protocols based on Real-time PCR including commercial assays with good sensitivity are now available for HEV diagnosis. A recent study compared five Real-time PCR procedures for HEV RNA detection with detection rates ranging from 83 to 100% [17]. In another study aiming at investigating the performance of molecular-based assays (conventional and Real Time PCR assay) using a panel of HEV-containing plasma samples, the authors found a marked difference in sensitivity between the assays, with the most sensitive methods being those based upon real-time RT-PCR [18]. Although real time PCR provides sensitive detection of HEV genome, it is not useful for molecular characterization and typing. Indeed, the length of the fragments amplified by real time PCR is usually less than 100 nucleotides and they are located on conserved parts of the genome to reach higher PCR efficacy and sensitivity [18]. Genotyping and phylogenetic analysis requires longer fragments within more variable regions. Conventional PCR followed by sequencing of PCR products can identify the genotype and, depending on the region, the subtype, thus helping in defining the origin of infection and tracing the source of contamination. However, the great genetic diversity of HEV viruses, classified into four major genotypes and several subtypes within each genotype, makes it very difficult to design sensitive universal primers. Several RT-PCR assays have been developed capable of detecting different HEV types, including those derived from animals. In this work we compared the performances of 5 widely used broad spectrum methods targeting the ORF1, ORF2, and ORF2/3 regions, using a collection of positive serum samples previously confirmed as HEV-positive by anti-HEV IgM and IgG assays as well as by Real-Time PCR [9]. In total, only 46% of serum samples tested positive for HEV RNA by nested PCR, combining the results obtained with the 5 assays. The conventional PCR assays were less sensitive than the Real-Time assay, being able to detect only a portion of the positive samples. It should be noted, however, that a comparison of the sensitivity of Real-Time PCR vs. conventional PCR was out of the scope of this study; indeed the quantitative and qualitative analyses were not performed simultaneous, on the same extracted RNAs. Serum samples were collected from 2004 to 2013 in Italy and analyzed immediately after collection, by a diagnostic strategy based on genomic (Real-Time) and serological assays [9]. They were then stored at -70°C until the genome extraction for this study; the newly extracted RNAs were then aliquoted and thawed only once. Thawing of serum and RNAs are possible causes of HEV RNA degradation and could partly explain the lower sensitivity of PCR assays even if a correlation between time of storage and performances of PCR assays was not observed.

In samples found positive by both quantitative and qualitative PCR, we found, in general, a positive trend between the yield of RT-PCR positivity and the viral load. However, some samples were not detected by the nested assay despite the high viral load; on the other hand, samples with low RNA quantity were detected. In these cases, the sensitivity of the nested PCR assays was not linked to the viral load, therefore likely depending on the genetic variability of the strains. Eleven samples were positive for HEV-RNA by one or more of the above assays: 9 were positive for G1 (imported) and two for G3 (authoctonous). The countries from which HEV cases seem to have been imported are Bangladesh, India, and Thailand, in agreement with previous Italian studies [6,7]. Phylogenetic analysis showed a match between sequences derived from patients with travel-related HEV and sequences from the geographical regions in which infection was acquired. The two HEV G3-positive patients had not travelled outside Italy; unfortunately risk factors for these infections have not been investigated. Sequences from patients with autochthonous HEV clustered on the same branch with published swine HEV isolates, which are thought to play a role in the transmission of HEV. It is noteworthy that they also clustered with HEV sequences detected in sewage and river samples in Italy. In industrialized countries HEV has been detected in different water environments [19]; moreover, infectious particles have been reported to occur in sewage, indicating the existence of a potential public health risk from the contamination of surface water with HEV [20].

Analysis of the relationship between the genotype and the viral load showed that HEV G1 (imported, travellers) infections use to display a higher viral load than HEV G3 (authoctonous, non-travellers) infections. The overall yield of RT-PCR testing found among samples from travellers was twice higher than the yield obtained among the non-travellers. Differences in viral concentration may possibly be linked with the severity of symptoms; indeed, in a previous study, we found that the course and outcome of clinical illness in patients developing travel-related HEV were different from those observed in patients developing autochthonous HEV. Patients from the first group were all hospitalized due to severe symptoms; the second group of patients, on the other hand, showed less severe symptoms and did not require hospitalization [6,7].

As for the performance of the different assays, Method A which targets the MTase gene displayed a marked higher analytical sensitivity than the other assays, with 8/11 positivities detected; moreover it detected both G1 and G3 HEV strains. Method B, which targets the RdRp detected only 3 out of 11 positive samples. This method was chosen for this study since it was successfully used recently for the identification of HEV in symptom-free migrants and environmental samples in Italy [21]; in the present study the sensitivity was low, despite having attempted to improve PCR sensitivity using different RNA dilutions or PCR conditions (data not shown). However, it was able to detect two additional G1-positive samples (not detected by Method A). Considering both assays, therefore the ORF1 region seems to be the most suitable for HEV genotype identification, with 10/11 positivities detected. However the ORF1 assays are not useful for subtype characterizations which will require the sequencing of the capsid region. The capsid assays (Methods C and D) were also found to be less sensitive than the ORF2/3 assay; similar results were obtained by Inoue and coworkers who found the ORF2/3 (Method E) to be two to three times more sensitive than ORF2 PCR (Method C) [8].

Although the higher sensitivity of Real-Time assays makes them more promising for diagnostic use, it is important to note that they are not useful for sequencing so the improvement of conventional RT-PCR assays is still needed to obtain information on molecular epidemiology of HEV.

## Conclusions

In conclusion, results from this work, in agreement with previously published studies [7,9], confirm that in Italy, although autochthonous cases do occur, HEV is predominantly travel-related. The variability of conventional PCR assay sensitivity suggests the need for the improvement and standardization of RT-PCR assays as they are an essential tool for molecular characterization of HEV.

## Abbreviations

HEV: Hepatitis E virus; G1: Genotype 1; G2: Genotype 2; G3: Genotype 3; G4: Genotype 4; ORF: Open reading frame; RdRp: RNA-dependent RNA polymerase; MCMC: Markov Chain Monte Carlo; MTase: Methyltransferase.

## Competing interests

The authors declare that they have no competing interests.

## Authors’ contributions

This study was conceived by GLR and ARC. All authors contributed to carry out the experiments, to the interpretation of the data, reviewed the manuscript critically and approved the final version.
